# Ugonin P facilitates chondrogenic properties in chondrocytes by inhibiting miR-3074-5p production: implications for the treatment of arthritic disorders

**DOI:** 10.7150/ijbs.108789

**Published:** 2025-01-21

**Authors:** Ting-Kuo Chang, Trung-Loc Ho, Yen-You Lin, Le Huynh Hoai Thuong, Kuan-Ying Lai, Chun-Hao Tsai, Chih-Chuang Liaw, Chih-Hsin Tang

**Affiliations:** 1 Department of Medicine, Mackay Medical College, New Taipei, Taiwan.; 2 Division of Spine Surgery, Department of Orthopedic Surgery, MacKay Memorial Hospital, New Taipei, Taiwan.; 3 Graduate Institute of Biomedical Sciences, China Medical University, Taichung, Taiwan.; 4 Translational Medicine Center, Shin Kong Wu Ho-Su Memorial Hospital, Taipei, Taiwan.; 5 Department of Marine Biotechnology and Resources, National Sun Yat-sen University, Kaohsiung, Taiwan.; 6 Department of Sports Medicine, College of Health Care, China Medical University, Taichung, Taiwan.; 7 Department of Orthopedic Surgery, China Medical University Hospital, Taichung, Taiwan.; 8 Graduate Institute of Natural Products, Kaohsiung Medical University, Kaohsiung, Taiwan.; 9 Department of Pharmacology, School of Medicine, China Medical University, Taichung, Taiwan.; 10 Chinese Medicine Research Center, China Medical University, Taichung, Taiwan.; 11 Department of Medical Laboratory Science and Biotechnology, College of Medical and Health Science, Asia University, Taichung, Taiwan.

**Keywords:** Arthritis, *Helminthostachys zeylanica*, ugonin P, miR-3074-5p, Aggrecan, type II collagen

## Abstract

Arthritis is a chronic inflammatory disease that causes joint damage, with osteoarthritis (OA) and rheumatoid arthritis (RA) being the most common types. Both conditions are characterized by cartilage degradation due to an imbalance between repair and breakdown processes. Chondrocytes, the key cells in articular cartilage, maintain its structure by producing an extracellular matrix rich in aggrecan and type II collagen (COL2). MicroRNAs (miRNAs), small noncoding RNAs, regulate genes critical for cartilage balance and are involved in the progression and treatment of OA and RA. Recently, herbal medicines have gained attention for arthritis treatment. Ugonin P, a flavonoid from *Helminthostachys zeylanica Hook*, is known for its antioxidant and anticancer effects, but its role in cartilage homeostasis is unclear. This study explores ugonin P's chondrogenic effects and its molecular mechanisms involving miRNA regulation. Analysis of Gene Expression Omnibus (GEO) data and clinical samples revealed reduced aggrecan and COL2 levels in OA and RA, while miR-3074-5p levels were elevated, suppressing these proteins. Ugonin P, without affecting cell viability, enhanced aggrecan and COL2 production and promoted chondrocyte differentiation by downregulating miR-3074-5p and activating MAPK pathways. These findings suggest ugonin P as a promising therapeutic candidate for arthritis management.

## 1. Introduction

Pain, stiffness, a reduction in range of motion, and joint abnormalities are some of the symptoms of arthritis, a condition that inflames and hurts the joints 1, 2. Rheumatoid arthritis (RA) and osteoarthritis (OA), which afflict millions of individuals globally, are the two most frequent types of arthritis 3, 4. The primary difference between OA and RA lies in the underlying cause of the joint symptoms. OA results from mechanical wear and tear on the joints, while RA is an autoimmune disorder in which the body's immune system mistakenly assaults the joints 5. However, both conditions share similar characteristics, such as cartilage degradation and joint stiffness, which are often linked to age-related disruptions in the balance between cartilage degradation and repair 6, 7. For yet, OA and RA have no known remedy. The goals of current treatments are to lessen joint functional capability and pain and discomfort. Consequently, there is an unfulfilled demand for the creation of innovative OA and RA management techniques.

Articular cartilage, a smooth and viscoelastic tissue, is specifically designed to bear and distribute loads across diarthrodial joints. Despite its unique mechanical properties, articular cartilage has limited regenerative capacity 8, 9. Chondrogenesis is fundamental for the development of chondrocytes during embryogenesis and for skeletal repair in adulthood 10. This process initiates with the aggregation and condensation of loose mesenchymal cells. In this early stage, the condensing mesenchyme produces various extracellular matrix (ECM) and cell adhesion molecules. Among ECM components, type II collagen (COL2), the predominant collagen produced by chondrocytes and a key structural component of articular cartilage, shows a progressive decline in expression with advancing age 11. On the other hand, aggrecan, the major proteoglycan in articular cartilage, is critical for its correct functioning 12. Chondrocytes, the cells embedded within the cartilage matrix, play a crucial role in maintaining the tissue by proliferating and producing an ECM enriched with aggrecan and COL2, both of which are essential for proper cartilage function 13. These cells respond to external stimuli and tissue damage, contributing to the development of degenerative conditions. Therefore, enhancing chondrocyte homeostasis has become a key focus that cannot be ignored in the development of arthritis treatment strategies.

Small, highly conserved non-coding RNA molecules called miRNAs control the expression of certain genes. Their role in arthritis development has gained significant attention, with growing evidence highlighting the aberrant synthesis of numerous miRNAs in both clinical tissues and experimental models of OA and RA 14, 15. Recently, miRNAs have been recognized as crucial regulators of cartilage homeostasis 16. The breakdown of the articular cartilage matrix in OA chondrocytes is induced by the suppression of miR-140 synthesis by IL-1β and TGF-β, which also upregulate the expression of catabolic factors 17. In RA, miR-146a diminished the inflammatory reaction by downregulating the NF-κB signalling 18. Investigating these miRNAs offers novel mechanistic and therapeutic insights into the progression of arthritic disorders.

Due to their low toxicity and biological activity, both natural compounds and synthetic products derived from natural prototypes have garnered significant highlight 19, 20. Ugonin-derived compounds (A-U), isolated from the herbal plant *Helminthostachys zeylanica* (*H. zeylanica*), have garnered interest among researchers for their pharmacological properties. Research has revealed that neougonin A suppresses inflammatory responses in macrophages triggered by lipopolysaccharide 21. Additionally, ugonin J and K exhibit antidiabetic properties through the inhibition of α-glucosidase 22. Ugonin M has been identified for its hepatoprotective capabilities 23, whereas ugonin U demonstrates immunomodulatory effects on human neutrophils 24. Ugonin P, a natural flavonoid compound derived from the root and rhizome of *H. zeylanica*, is known for its anti-inflammation, anti-redox, and anti-tumor properties 25-27. In skeletal diseases, ugonins have been shown to inhibit RANKL-induced osteoclastogenesis and induce osteoclast apoptosis, highlighting their potential as therapeutic agents for bone loss conditions 28. However, their effects on chondrogenic properties remain unknown. Here, we report that ugonin P promotes aggrecan and COL2 synthesis and facilitates chondrogenic properties in chondrocytes by inhibiting miR-3074-5p expression. Ugonin P could serve as the basis for developing novel therapeutic agents for the management of arthritic disorders.

## 2. Materials and methods

### 2.1. Materials

The procedure described in an earlier document 25 was followed in the preparation of Ugonin P. From Sigma-Aldrich (St. Louis, Missouri, USA), 3-(4,5-dimethylthiazol-2-yl)-2,5-diphenyltetrazolium bromide (MTT) was acquired. Cell culture materials were obtained from Gibco-BRL (Grand Island, NY). The comprehensive origin of primers, antibodies, pharmacological inhibitors and siRNA were listed in Supplementary Table 1-4.

### 2.2. Cell cultures

The mouse chondrocytic cell line ATDC5 was kindly provided by Dr. Shyh-Ming Kuo (I-Shou University, Kaohsiung, Taiwan) and maintained in a 1:1 mixture of DMEM and Ham's F12 medium containing 17.5 mM glucose and supplemented with 5% FBS, 100 U/mL penicillin and 100 µg/mL streptomycin, in a humid atmosphere containing 5% CO_2_ at 37 °C 29.

From resected cartilage specimens acquired from primary total knee arthroplasty procedures, human articular chondrocytes were separated 30, 31. Chondrocytes were separated by sequential enzymatic processing at 37 °C using 0.1% hyaluronidase for 30 min and 0.2% collagenase for 1 hr after the cartilage pieces were finely chopped. 70 μM nylon filters were used to filter the isolated chondrocytes. The cells were cultivated and developed in accordance with earlier publications 30, 31. Human primary chondrocytes were cultured in DMEM supplemented with 20 mM HEPES, 10% FBS, 2 mM L-glutamine, 100 U/mL penicillin, and 100 µg/mL streptomycin. Cells were utilized for experiments between the second and sixth passages.

### 2.3. Bioinformatics analysis

A dataset GSE55235 was obtained from the Gene Expression Omnibus (GEO) database and used to investigate the levels of aggrecan and COL2 32. We used QIAGEN Ingenuity Pathway Analysis (IPA) (Hilden, Germany) to investigate canonical signaling pathways. The single-cell gene expression analysis of human cartilage, obtained from the GEO database (ID: GSE255460), was reanalysed to examine the expression of aggrecan and COL2 genes in cartilage from non-arthritis and arthritis patients using Loupe Browser 8 software.

The open-source software libraries miRWalk 3.0 (http://mirwalk.umm.uni-heidelberg.de/) searched to investigate miRNAs that potentially bind with aggrecan and COL2 33. The miRWalk prediction process involved several steps:

Step 1: Target Mining: In the Target Mining section, the “Genes” option was selected, and the species (“mouse” or “human”) was specified. The corresponding Entrez gene IDs, such as 176 for ACAN and 1280 for COL2A1, were entered. Subsequently, the “Submit” and “Processed” buttons were clicked to initiate the analysis.

Step 2: Database Selection: Users were redirected to a new page where specific databases, including TargetScan, miRDB, and mirtarbase, could be selected. The 3'UTR option was also chosen to refine the search criteria.

This process generated a ranked list of predicted miRNAs that bind to the 3'UTR regions of ACAN and COL2A1. Higher scores indicated greater agreement across the databases regarding miRNA binding to the target gene.

Level of miRNA expression were examined through miRNA sequencing from the GEO database (GEO: GSE143514, GSE175962, GSE124373).

### 2.4. MTT assay

Cells were either treated with or without different doses of ugonin P for a whole day. The MTT buffer was added and dissolved in dimethylsulfoxide at a concentration of 0.5 mg/mL. BioTek (Winooski, Vermont, USA) produced the microplate reader that was used to test the absorbance at 570 nm.

### 2.5. Quantitative real-time PCR

Total RNAs were prepared using the manufacturer's method for TRIZOL reagent (MDBio, Inc., Taipei, Taiwan). The isolated RNA samples were transcribed to cDNA with the utilization of M-MLV RT kit (Thermo Fisher Scientific, Waltham, MA, USA) according to the manufacturer's protocol. Expression levels of miRNA were quantified using the Mir-X™ miRNA First-Strand Synthesis kit (Terra Bella Avenue, Mountain View, CA, USA). The expression of mRNA and miRNA was measured by using qPCR in the StepOnePlus™ Real-Time PCR System from Applied Biosystems 34, 35. Glyceraldehyde 3-phosphate dehydrogenase (GAPDH) and U6 were used as housekeeping gene controls to normalize mRNA and miRNA expression, respectively.

### 2.6. RNA sequencing

ATDC5 cells were incubated with 3 μM of ugonin P for 24 hrs. The isolated RNA samples were used for following library preparation. Then libraries with different indexs were multiplexed and loaded on an Illumina HiSeq/ Illumina Novaseq/ MGI2000 instrument (Illumina, San Diego, CA, USA) for sequencing using a 2×150 paired-end configuration according to manufacturer's instructions. Differential expression analysis used the DESeq2 Bioconductor package. We utilized a |log_2_fold change (FC)| > 2 with adjusted *p* value < 0.05 as the threshold for significantly differential expression. Kyoto Encyclopedia of Genes and Genomes (KEGG) is the primary public pathway database used in this analysis.

### 2.7. Western blot analysis

Cells were lysed in 100 µL RIPA lysis buffer supplemented with a protease inhibitor cocktail (Roche, Indianapolis, IN, USA). Equal concentrations of total protein (30μg) were run on SDS-PAGE gels and then electrotransferred to PVDF membranes (Millipore, Bedford, MA, USA). Membranes were blocked in 5% non-fat milk in Tris-buffered saline for 1 hr and incubated with primary antibodies overnight at 4°C. Thereafter, the membranes were washed with TBST and incubated at room temperature for 1 hr with appropriate HRP-conjugated secondary antibodies. Detection of target proteins was performed by adding ECL reagents (Merck-Millipore) followed by documentation through autoradiography 36, 37.

### 2.8. Alcian Blue staining

For differentiation induction, ATDC5 cells (5 × 10⁴ cells/well) were seeded into 12-well plates. Once the cells reached confluence, they were treated with a chondrogenic differentiation medium. This medium consisted of growth medium supplemented with 1% Insulin-Transferrin-Selenium solution (1 mg/mL insulin, 0.55 mg/mL transferrin, 0.67 mg/mL sodium selenite) (Gibco, Cat. No. 41400045) and 50 µg/mL L-ascorbic acid 2-phosphate (MCE, Cat. No. HY-103701). Cells were differentiated for 14 days, during which each medium was replaced every 2 to 3 days. Cells were then fixed in 4% formaldehyde for 30 mins and stained with 1% Alcian Blue 8GX (Sigma Alrich, Cat. No. A5268-10G). The cells were then washed in diH₂O three times and applied with 6 mol/L guanidine for 2 hrs. The OD was measured at 620 nm while the cells were observed under an inverted microscope and pictures captured.

### 2.9. Cell transfection

The cells (5 × 10⁵ cells/well) were seeded into 6-well plates, and then various miRNA mimic or miRNA inhibitors and pharmacological siRNAs were transfected into cells with Lipofectamine 2000 transfection reagent (Waltham, Massachusetts, USA) following manufacturer's instructions.

### 2.10. Statistics

Statistical analyses were performed using GraphPad Prism 8.2 software. Statistical comparisons of more than three groups were performed using one-way analysis of variance (ANOVA) with Bonferroni's post hoc test, and two-way ANOVA was used with more than two factors. Results are expressed as the mean ± standard deviation (SD). *p* < 0.05 was considered statistically significant.

## 3. Results

### 3.1 Downregulation of aggrecan and COL2 levels in OA and RA patients

**Figure 1:**
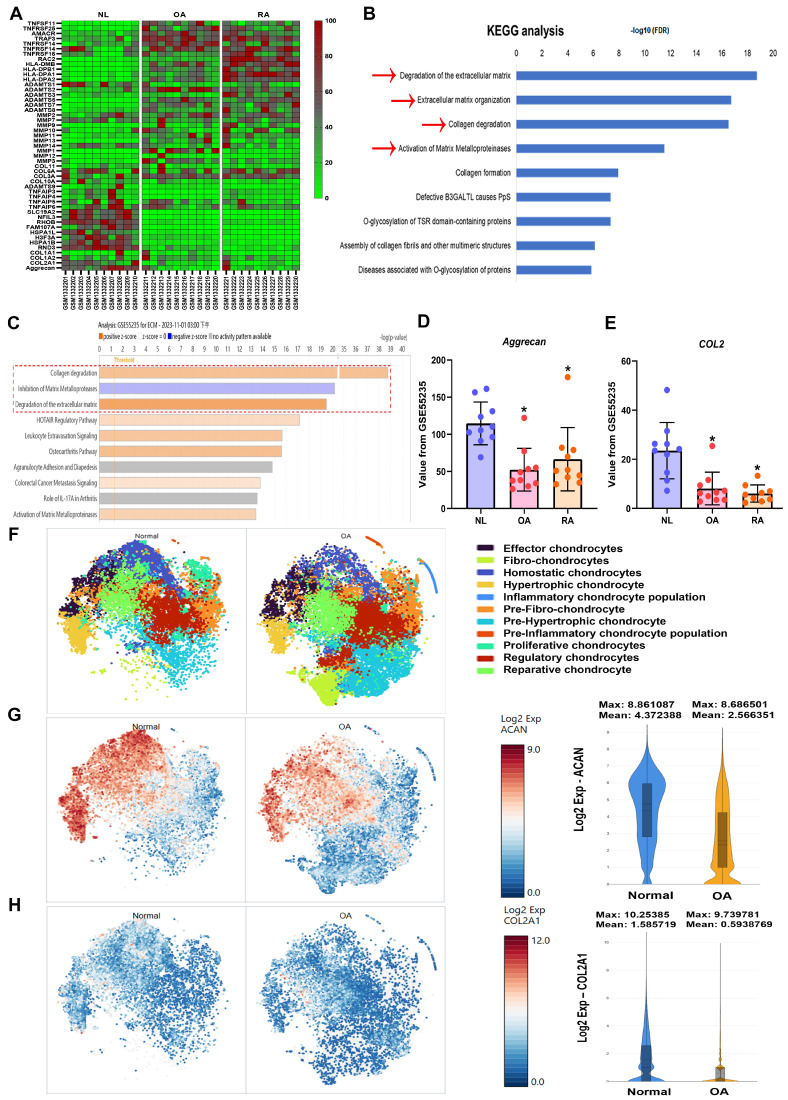
** High cartilage degradation gene profile and reduced aggrecan and COL2 levels in OA and RA patients.** (A) Heatmap showing differentially expressed ECM and cartilage degradation genes from the GSE55235 dataset. (B&C) KEGG and IPA pathway enrichment figure showing pathways that were significantly changed in the GSE55235 dataset. (D&E) Aggrecan and COL2 gene levels in normal, OA and RA patients retrieved from the GEO dataset. (F) Annotation of cell types in human cartilage tissues from GSE55460 dataset. (G&H) The distribution of aggrecan and COL2 gene expression loci was analysed across different cell types in human cartilage, comparing normal and arthritis patients using single-cell gene expression data. *p < 0.05 compared with the normal controls.

Synovial inflammation, cartilage degradation, and bone breakdown are common characteristics in the pathogenesis of OA and RA 38, 39. To further confirm these features in clinical patients, the GEO database was analyzed. The GSE55235 dataset revealed numerous differences in gene expression between normal individuals and arthritis patients (Fig. 1A). KEGG and IPA analyses of the GEO database showed that biological functions such as collagen degradation, ECM breakdown, and activation of matrix metalloproteinases are enriched in OA and RA (Fig. 1B&C). Among the top biological functions, it was found that cartilage components including aggrecan and COL2 are significantly lower in both OA and RA patients compared to controls (Fig. 1D&E). To enhance our understanding of the roles of aggrecan and COL2 in arthritis, we analysed the publicly available single-cell RNA sequencing dataset (GSE55460), comprising samples from normal human cartilage tissues and cartilage tissues from patients with arthritis (Figure 1F). The analysis revealed a reduced distribution and expression of aggrecan and COL2 genes in the cartilage tissues of arthritis patients (Figure 1G-H). These results indicate that both OA and RA patients exhibit ECM degradation and reduced levels of cartilage matrix components, including aggrecan and COL2.

### 3.2 Ugonin P treatment promotes aggrecan and COL2 expression and enhances chondrogenesis

**Figure 2:**
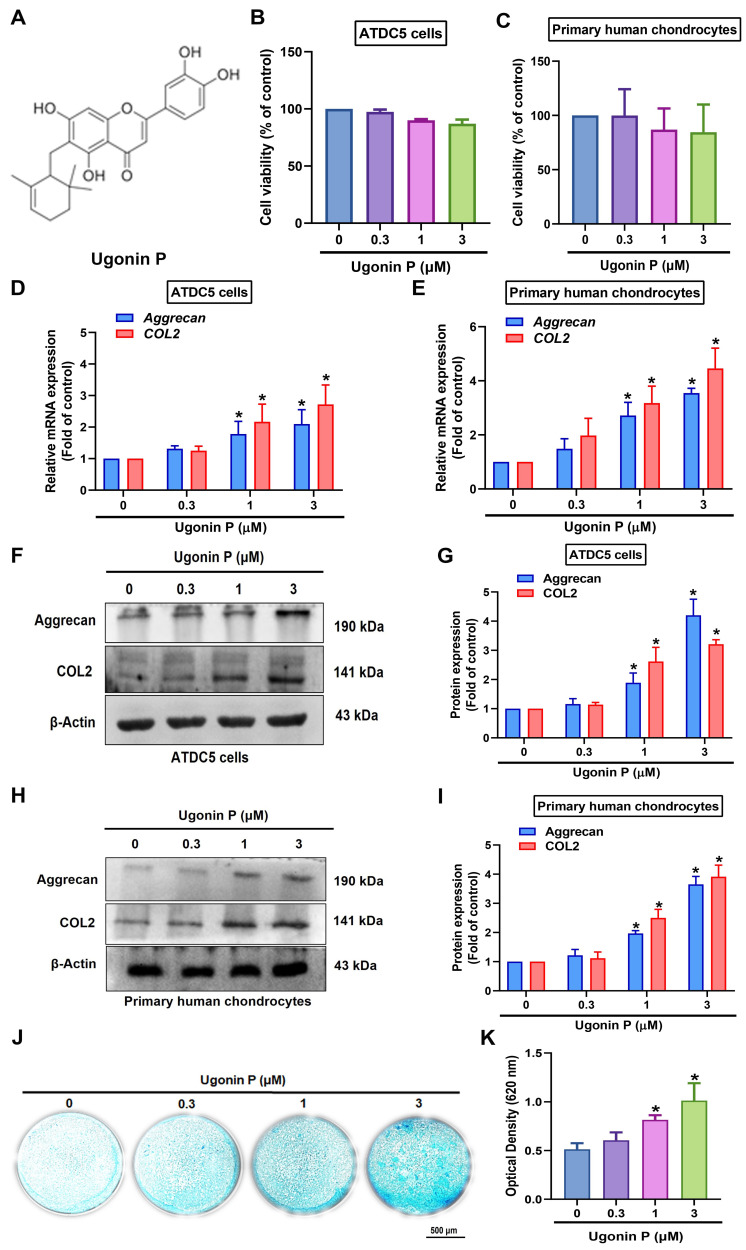
** Ugonin P induces aggrecan and COL2 production and enhances chondrogenesis.** (A) The chemical structure of ugonin P. (B&C) Cells were treated with different concentrations of ugonin P (0.3 μM, 1 μM, and 3 μM) for 24 hrs, MTT assay was used to evaluate the cytotoxicity effect. (D-I) Cells were treated with ugonin P, the aggrecan and COL2 mRNA and protein expression was examined by qPCR and Western blot. (J&K) ATDC5 cells were cultured with chondrogenic differentiation medium and treated with ugonin P for 2 weeks. Alcian blue staining was performed and quantified; scale bar = 500 μm. *p < 0.05 compared with the control group.

Ugonin compounds have been shown to significantly impact anti-resorptive diseases, such as osteoporosis and osteolytic bone metastasis 28. The chondrocytic cell line ATDC5 and human primary chondrocytes were used to examine the chondrogenic properties of ugonin P (Fig. 2A). MTT assay results indicated that ugonin P did not affect the viability of either cell type (Fig. 2B&C). Stimulation of cells with ugonin P markedly augmented the mRNA and protein production of aggrecan and COL2 (Fig. 2D-I). Next, Alcian blue staining was performed to track the formation of cartilaginous matrices, investigating the effect of ugonin P in chondrogenesis. Following ugonin P treatment, Alcian blue staining intensity increased in a concentration-dependent manner (Fig. 2J&K). These findings indicate that ugonin P increases the synthesis of aggrecan and COL2 in chondrocytes, thereby enhancing their chondrogenic potential.

### 3.3 miR-3074-5p regulates ugonin P-augmented aggrecan and COL2 expression, as well as chondrogenesis

**Figure 3:**
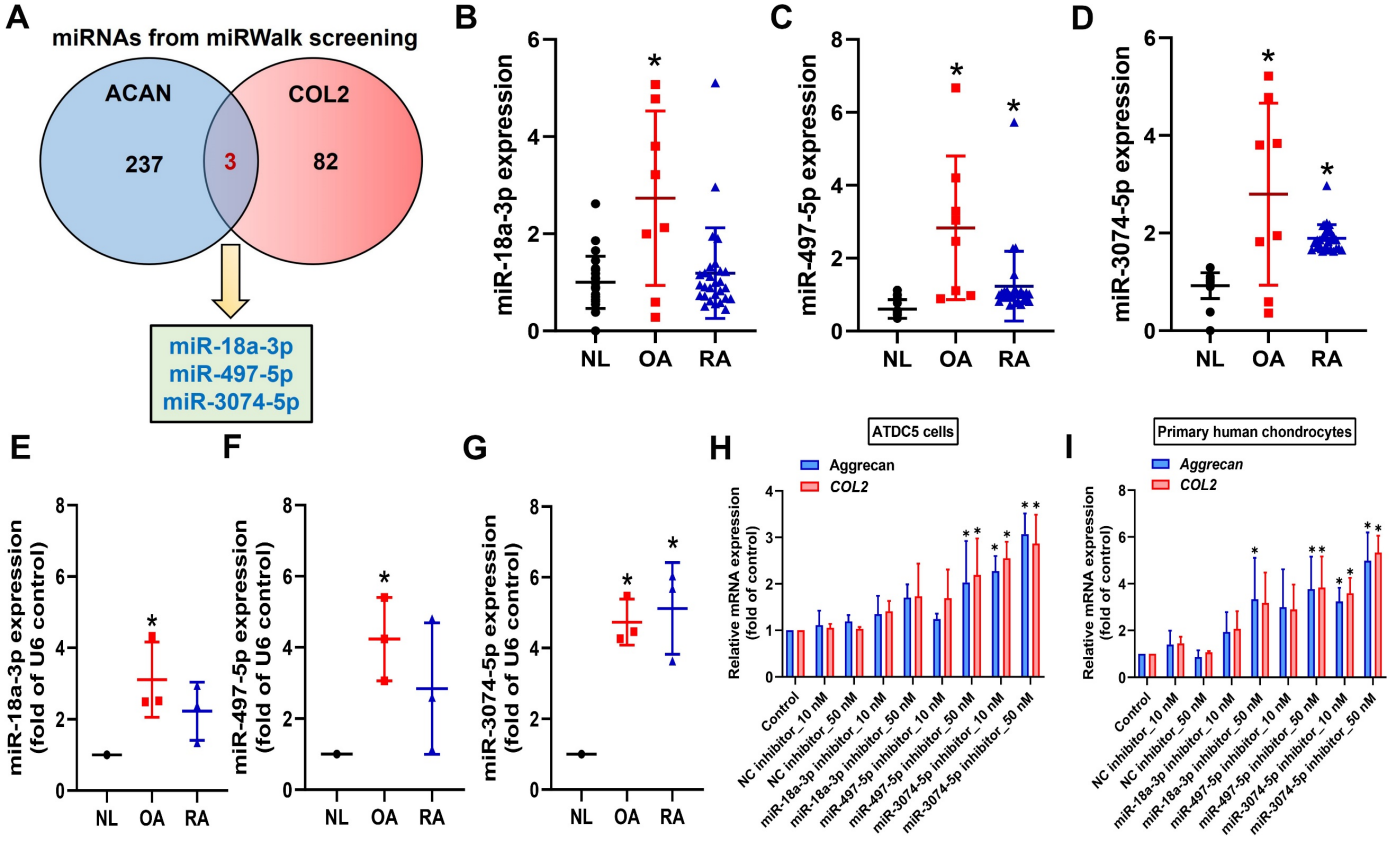
** miR-3074-5p controls aggrecan and COL2 expression.** (A) miRWalk identified miR-18a-3p, miR-497-5p, and miR-3074-5p as directly binding to the 3' UTRs of both aggrecan and COL2. (B-D) miRNAs levels in normal, OA and RA patients retrieved from the GEO dataset. (E-G) The miRNAs levels in normal, OA and RA patients was examined by qPCR. (H&I) Cells were transfected with indicated miRNA inhibitors, the aggrecan and COL2 mRNA expression was examined by qPCR. *p < 0.05 compared with the control group.

miRNAs inhibit mRNA translation by binding to the 3' UTR of target mRNA and participate in the management of biological pathways 40. Using miRWalk bioinformatics software, we identified miRNAs that directly bind to the 3' UTRs of both aggrecan and COL2 and found only three: miR-18a-3p, miR-497-5p, and miR-3074-5p (Fig. 3A). To investigate clinical relevance, analysis of GEO datasets revealed that miR-497-5p and miR-3074-5p were markedly upregulated in OA and RA patients (Fig. 3B-D). However, our clinical data indicated that only miR-3074-5p, and not miR-18a-3p or miR-497-5p, was significantly upregulated in both OA and RA patients (Fig. 3E-G). Transfection with miR-3074-5p inhibitor, but not miR-18a-3p or miR-497-5p inhibitors, enhanced aggrecan and COL2 synthesis in both ATDC5 cells and human primary chondrocytes (Fig. 3H&I). Therefore, miR-3074-5p is a critical mediator in controlling aggrecan and COL2 production during arthritis.

**Figure 4:**
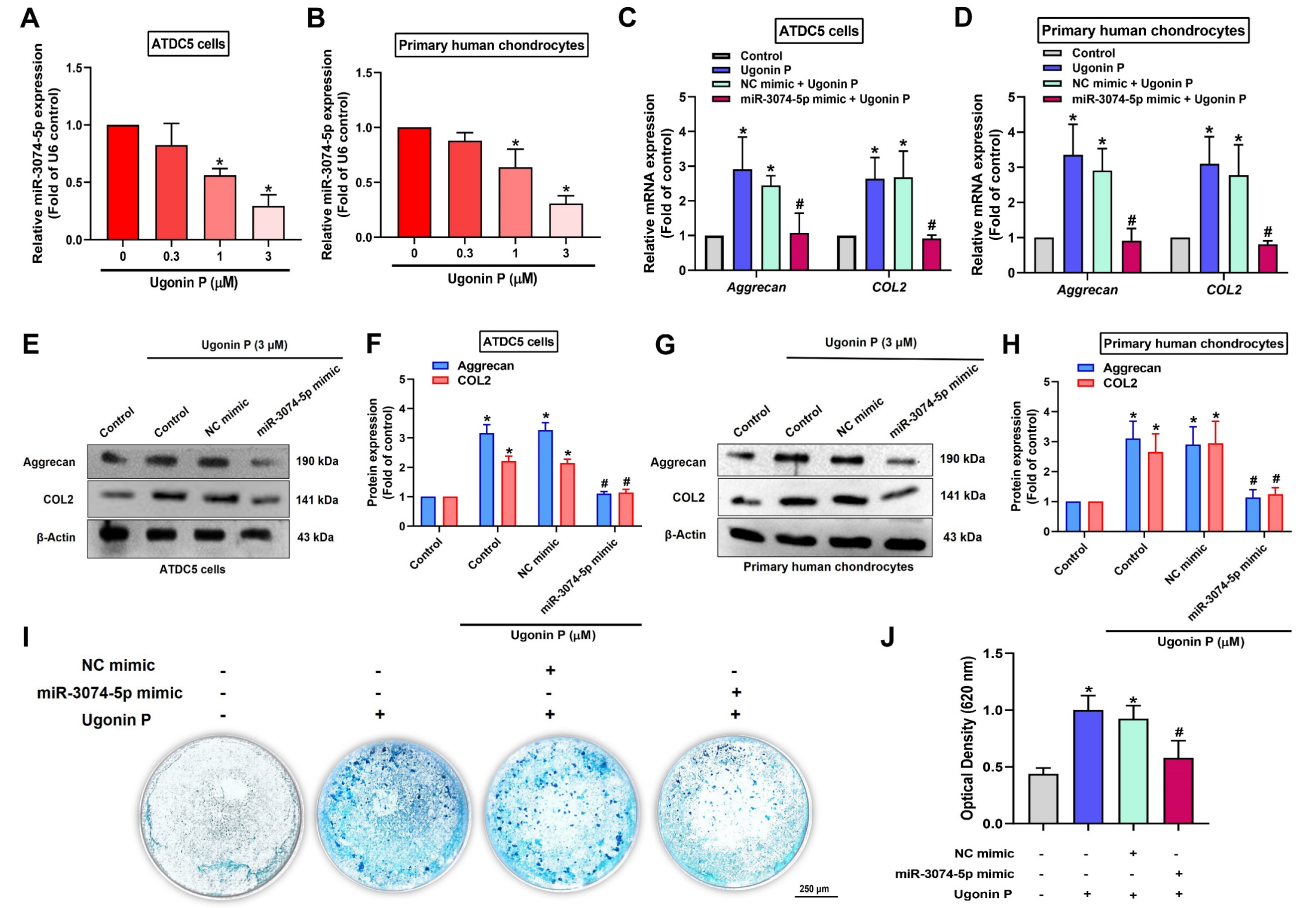
** Ugonin P enhances chondrogenic ability through inhibiting miR-3074-5p expression.** (A-B) qPCR analysis was performed to determine the expression levels of miR-3074-5p in ATDC5 cells and human primary chondrocytes following treatment with ugonin P at the indicated doses for 24 hrs. (C-H) Cells were transfected with a miR-3074-5p mimic (50 nM) and subsequently treated with ugonin P (3 μM), the aggrecan and COL2 mRNA and protein expression was examined by qPCR and Western blot. (I&J) ATDC5 cells were cultured with chondrogenic differentiation medium and treated with miR-3074-5p mimic (50 nM) and ugonin P (3 μM) for 2 weeks. Alcian blue staining was performed and quantified; scale bar = 250 μm. *p < 0.05 compared with the control group. ^#^*p* < 0.05 compared with the ugonin P-treated group.

We next investigated whether ugonin P promotes chondrogenesis by regulating miR-3074-5p. The results revealed that ugonin P inhibited miR-3074-5p production (Fig. 4A&B). The miR-3074-5p mimic antagonized ugonin P-induced aggrecan and COL2 synthesis (Fig. 4C-H). Furthermore, the miR-3074-5p mimic reversed ugonin P-promoted chondrogenesis (Fig. 4I&J), indicating that ugonin P induces aggrecan and COL2 expression, as well as chondrogenesis, by suppressing miR-3074-5p production.

### 3.4 Ugonin P enhances aggrecan and COL2 production in chondrocytes through the MAPK signaling pathway

**Figure 5:**
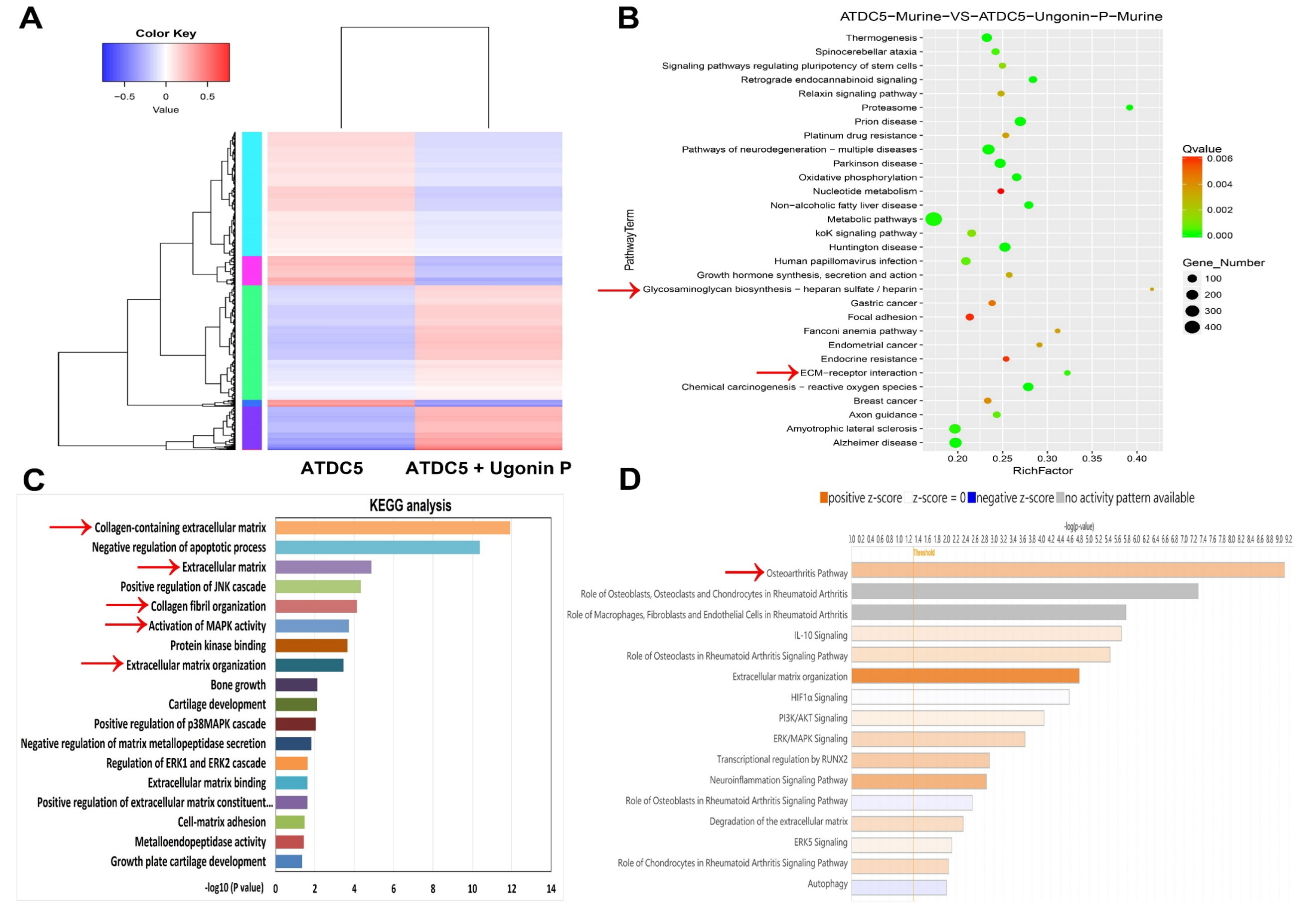
** RNA-sequencing analysis of the gene profile in chondrocytes treated with ugonin P.** (A) The cluster heatmap shows gene expression in ATDC5 cells compared to ATDC5 cells treated with ugonin P. (B-D) GO, KEGG and IPA enrichment analysis categorized the pathways that were significantly altered after ugonin P treatment.

**Figure 6:**
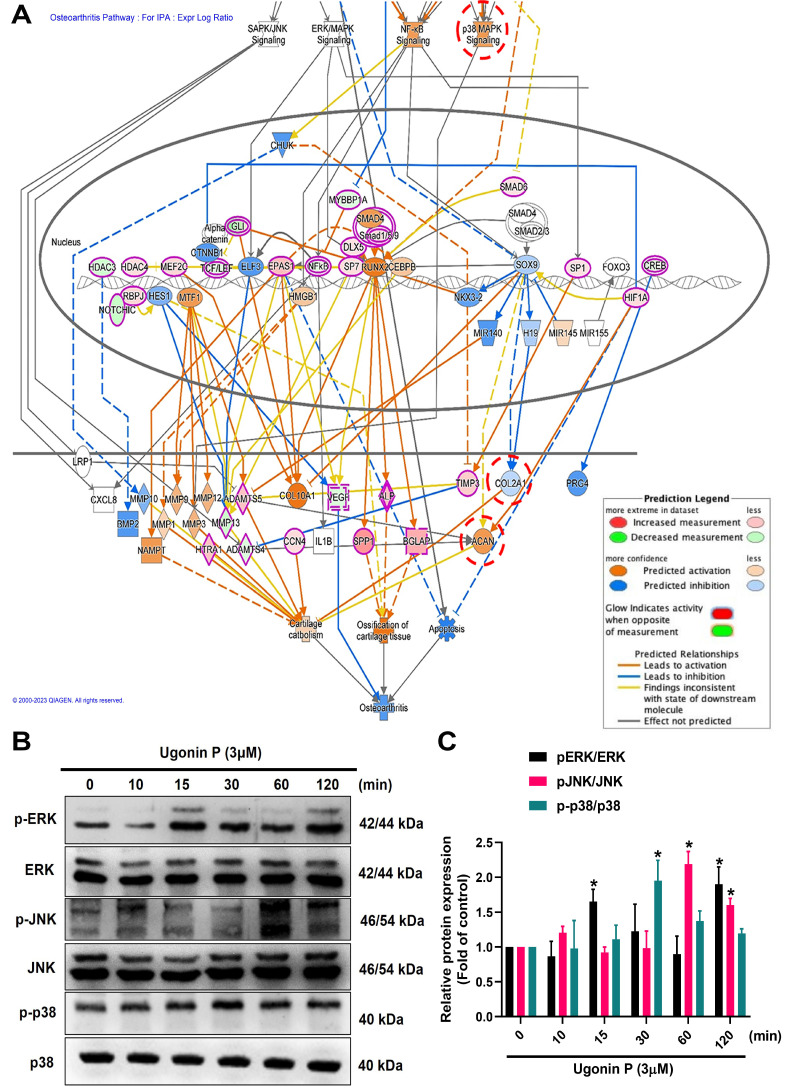
** Ugonin P activates MAPK pathway in chondrocytes.** (A) IPA analysis signaling pathways in ATDC5 cells compared to ATDC5 cells treated with ugonin P. (B&C) ATDC5 cells were treated with ugonin P (3 μM) for the indicated times up to 120 minutes, and the phosphorylation levels of ERK, p38, and JNK were assessed via Western blot analysis. Quantitative immunoblot results were analyzed using ImageJ software. *p < 0.05 compared with the control group.

**Figure 7:**
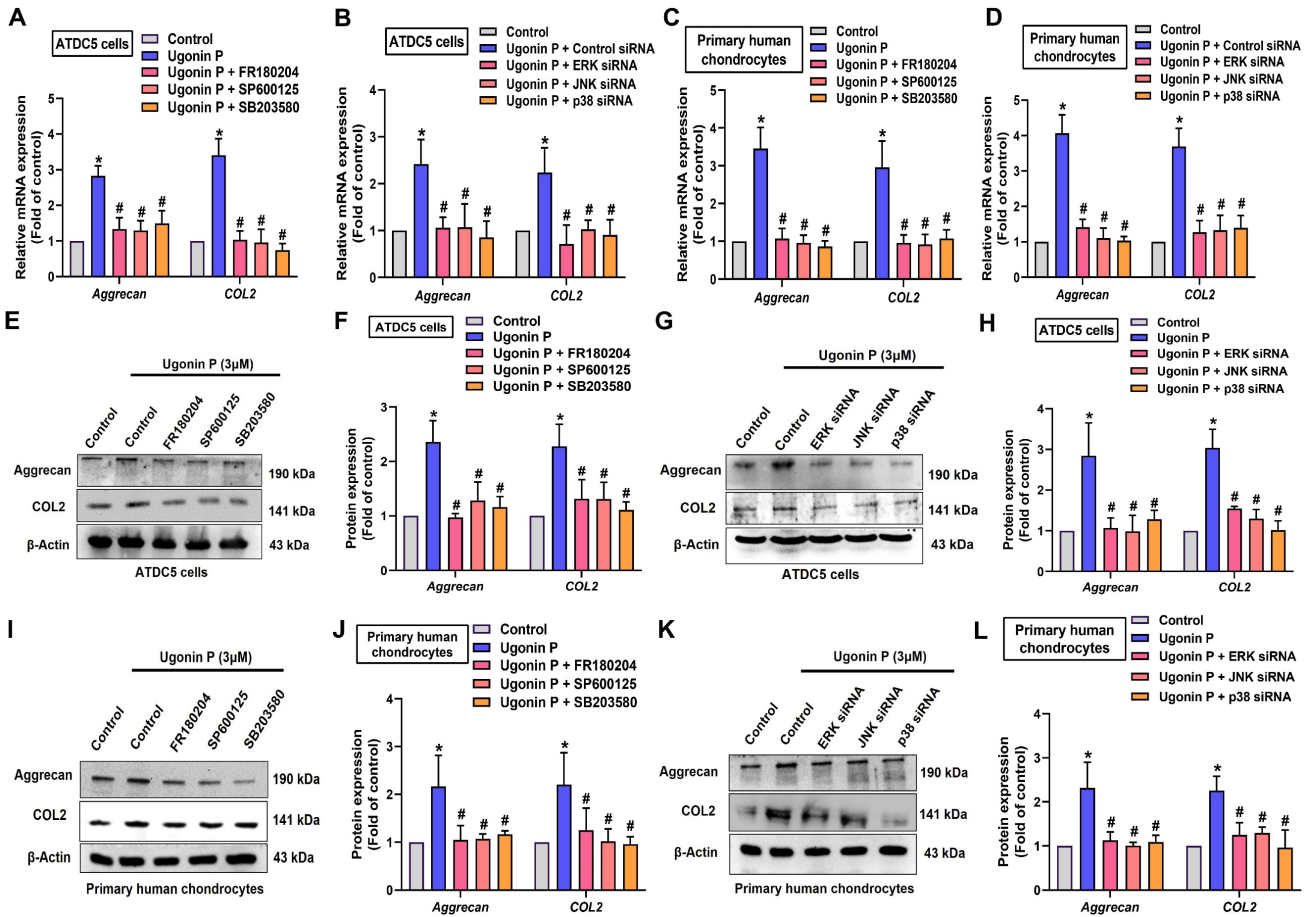
** Ugonin P enhances aggrecan and COL2 synthesis via MAPK pathway.** Cells were treated with ERK, p38 and JNK inhibitors or siRNAs then stimulated with ugonin P for 24 hrs. The aggrecan and COL2 expression was examined by qPCR and Western blot. *p < 0.05 compared with the control group. ^#^*p* < 0.05 compared with the ugonin P-treated group.

To identify the signaling pathways essential for ugonin P in regulating chondrogenesis, we performed RNA-sequencing analysis on ATDC5 cells with and without ugonin P treatment (Fig. 5A). GO and KEGG analysis indicated that ugonin P upregulated glycosaminoglycan biosynthesis, ECM organization, and MAPK signaling pathways (Fig. 5B&C). Additionally, IPA results highlighted a significant increase in osteoarthritis-related pathways, particularly those involving MAPK (Fig. 5D&6A). To validate these findings, we conducted western blot experiments to assess the role of MAPK signaling after ugonin P stimulation. The results found that ugonin P induced the phosphorylation of ERK, JNK, and p38 (Fig. 6B&C). Stimulation of cells with ERK (FR180204), JNK (SP600125), and p38 (SB203580) inhibitors or siRNAs reduced ugonin P-induced aggrecan and COL2 expression (Fig. 7). Thus, the MAPK signaling pathway is involved in ugonin P-facilitated aggrecan and COL2 expression in chondrocytes.

## 4. Discussion

Articular cartilage degradation is a hallmark shared by both OA and RA. Treatments for OA and RA that are now available, including as physical therapy, medication, and surgery, frequently fall short of expectations because they only address symptoms and do not effectively slow down or stop the biological processes that cause tissue destruction 41. Recent evidence suggests that alterations in the composition and physical properties of the ECM contribute to the development of OA and RA 42. The ECM is primarily composed of aggrecan and COL2, which are crucial for maintaining the normal physiological functions of cartilage 43. Our study, which analysed data from the GEO database and clinical samples, revealed that aggrecan and COL2 levels were significantly reduced in OA and RA patients. These findings indicate that strategies designed to enhance chondrogenic properties through the upregulation of aggrecan and COL2 expression may play a critical role in the regenerative therapy of OA and RA.

Chondrogenesis is primarily initiated by the condensation and subsequent differentiation of mesenchymal stem cells into chondrocytes, which are instrumental in synthesizing a cartilaginous ECM 44. Due to their rapid and extensive growth, homogeneity, and chondrogenic potential, ATDC5 cells are widely recognized as a robust *in vitro* model for studying chondrocyte differentiation 45-47. Here, ATDC5 cells were cultured in chondrogenic differentiation medium, and ugonin P promoted chondrogenesis as demonstrated by Alcian blue staining. Interestingly, transfection with a miR-3074-5p mimic, which targets aggrecan and COL2, reversed ugonin P-promoted chondrogenesis. Thus, ugonin P facilitates aggrecan and COL2 production and subsequently enhances chondrogenesis by inhibiting miR-3074-5p expression.

Small non-coding RNA molecules called miRNAs attach themselves to the 3'UTRs of mRNAs in order to cause degradation 48. miRNAs have been reported to play a crucial role in regulating anabolic and catabolic processes in articular cartilage, as well as mediating the inflammatory reaction and degenerative pathogenesis in RA and OA. In fact, previous studies have demonstrated that miR-20a suppresses chondrogenesis in ATDC5 cells 49, while miR-34a-5p is markedly upregulated in obese patients with late-stage OA, contributing to joint destruction 50. Through bioinformatic analysis, we suggested miR-3074-5p as a potential novel therapeutic target in OA and RA, as it directly binds to and suppresses the expression of aggrecan and COL2. Here, we revealed that miR-3074-5p expression levels were elevated in samples from OA and RA patients compared to normal samples. Furthermore, in ATDC5 cells and human primary chondrocytes, miR-3074-5p exhibited a stronger inhibitory effect on aggrecan and COL2 expression compared to miR-18a-3p or miR-497-5p. Notably, ugonin P markedly inhibits miR-3074-5p expression, thereby enhancing chondrogenic properties. Therefore, therapeutic inhibition of miR-3074-5p is a novel strategy to restore aggrecan and COL2 synthesis for managing arthritic disorders.

MAPK signaling pathways play a chief role in numerous cellular processes, such as apoptosis, differentiation, proliferation, and inflammation 51, 52. Previous studies have found that the MAPK pathway is involved in TGF-β-facilitated chondrogenesis by modulating the expression of cartilage-specific genes 53. Our RNA-sequencing results revealed significant gene changes after ugonin P stimulation. Osteoarthritis-related mechanisms, including ERK, JNK, and p38, emerged as top candidate signaling pathways in ugonin P-treated ATDC5 cells, according to IPA analysis. We found that ugonin P stimulation facilitates ERK, p38, and JNK phosphorylation. Incubating with ERK, p38, and JNK inhibitors blocked ugonin P-induced promotion of aggrecan and COL2 production. These results were confirmed using genetic siRNA, which showed similar effects in blocking ugonin P's chondrogenic ability. Therefore, activation of the MAPK pathway is a critical step in ugonin P-induced aggrecan and COL2 expression. Additionally, we examined the correlation between miR-3074-5p and the ERK, JNK, or p38 signaling pathways. The results, as detailed in Supplementary Table 5, indicate that miR-3074-5p can directly bind to the genes encoding ERK, JNK, and p38. Therefore, whether miR-3074-5p functions as an upstream molecule in ugonin P-mediated activation of ERK, JNK, and p38 pathways, and its role in chondrogenesis, requires further investigation.

Our findings demonstrate that ugonin P exerts a chondroprotective effect by inhibiting miR-3074-5p production and modulating key signaling pathways, including ERK, p38, and JNK. To advance the clinical translation of ugonin P, several critical steps are required. Comprehensive preclinical studies are necessary to evaluate its pharmacokinetics, bioavailability, and toxicity profile *in vivo*. Utilizing animal models, such as collagen-induced arthritis (CIA) or anterior cruciate ligament transection (ACLT) models, will provide essential insights into its therapeutic efficacy and safety. While ugonin P shows significant promise as a potential therapeutic agent for arthritis and cartilage-degenerative diseases, its clinical translation will require a systematic approach that addresses both efficacy and safety in preclinical and clinical stages. However, a limitation of our study is the inability to utilize the CIA or ACLT *in vivo* models to fully verify the role of ugonin P due to the limited quantity of the isolated compound. Future studies are necessary to investigate its chondrogenic protective effects in more detail using these models.

**Figure 8:**
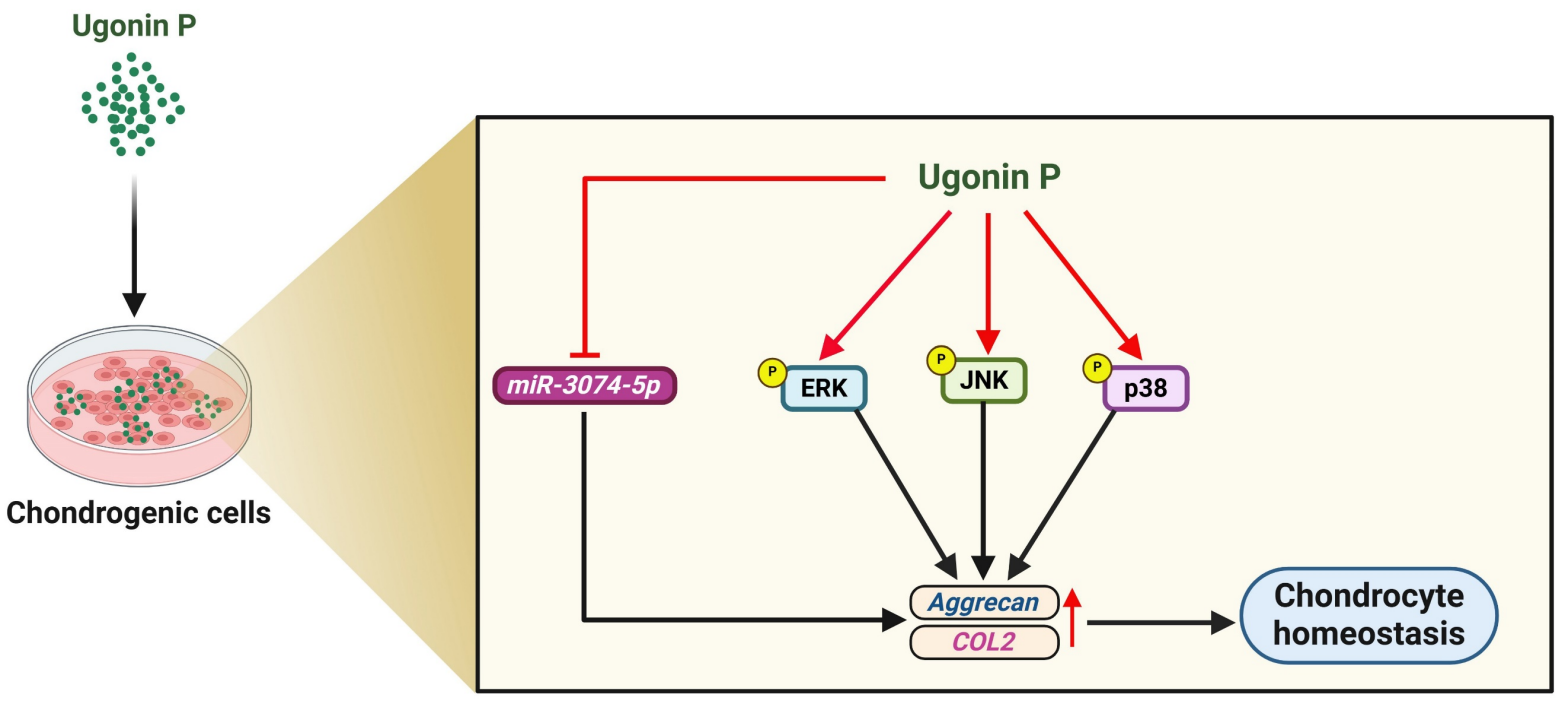
** Illustration depicting the effects of ugonin P on chondrogenesis. (The schema was generated utilizing BioRender.com).** Ugonin P induces aggrecan and COL2 production in chondrocytes and subsequently promotes chondrogenic properties by suppressing miR-3074-5p synthesis and activating MAPK pathways.

Pharmacotherapy has greatly benefited from the application of natural compounds and their structural analogs 54. *H. zeylanica* is a natural compound known for its anti-inflammatory, anti-cancer, anti-bone resorption, and hepatoprotective properties 23, 26, 28. However, the role of ugonin P in cartilage protection in arthritis remains unclear. In this study, we demonstrated that ugonin P promotes aggrecan and COL2 production in chondrocytes and subsequently enhances chondrogenic properties by suppressing miR-3074-5p synthesis and activating MAPK pathways (Fig. 8). We propose that ugonin P may be a novel therapeutic natural compound for treating arthritic disorders.

## Supplementary Material

Supplementary tables.

## Abbreviations

OA: osteoarthritis
RA: rheumatoid arthritis
COL2: type II collagen
ACAN: aggrecan
miRNAs: micro RNAs
MAPK: mitogen-activated protein kinase
ERK: Extracellular signal-regulated kinase
JNK: Jun N-terminal kinase
ECM: extracellular matrix
KEGG: Kyoto Encyclopedia of Genes and Genomes
IPA: Ingenuity Pathway Analysis
GEO: Gene Expression Omnibus
