# Supplementary material for: Ugonin P facilitates chondrogenic properties in chondrocytes by inhibiting miR-3074-5p production: implications for the treatment of arthritic disorders
Source: Int J Biol Sci. 2025 Jan 21;21(4):1378–90. doi: 10.7150/ijbs.108789 (PMC11844289; doi:10.7150/ijbs.108789)
Supplement: Supplementary file 1 — Supplementary tables. [file ijbsv21p1378s1.pdf]

Supplementary materials

Table 1: The primers were applied in this study

Gene	Species	Primers (5'-3')
Aggrecan	Human	Forward: CCCCTGCTATTTCATCGACCC<br>Reverse: GACACACGGCTCCACTTGAT
COL2	Human	Forward: TGGACGATCAGGCGAAACC<br>Reverse: GCTGCGGATGCTCTCAATCT
GAPDH	Human	Forward: ACCACAGTCCATGCCATCAC<br>Reverse: TCCACCACCCTGTTGCTGTA
Aggrecan	Mouse	Forward: GTGGAGCCGTGTTTCCAAG<br>Reverse: AGATGCTGTTGACTCGAACCT
COL2	Mouse	Forward: CCTCAAGGCAAAGTTGGTCCT<br>Reverse: CTCCCGTCTCACCGTCTTTT
GAPDH	Mouse	Forward: TGTGTCCGTCGTGGATCTGA<br>Reverse: TTGCTGTTGAAGTCGCAGGAG
hsa-miR-18a-3p	Human	ACTGCCCTAAGTGCTCCTTCTGG
hsa-miR-497-5p	Human	CAGCAGCACACTGTGGTTTGT
hsa-miR-3074-5p	Human	GTTCCTGCTGAACTGAGCCAG
mmu-miR-18a-3p	Mouse	ACTGCCCTAAGTGCTCCTTCTG
mmu-miR-497-5p	Mouse	CAGCAGCACACTGTGGTTTGTA
mmu-miR-3074-5p	Mouse	GTTCCTGCTGAACTGAGCCAGT

Table 2: The antibodies of WB applied in this study

Antibodies	Source	Catalog no	Working concentrations
Aggrecan	Abcam, Waltham, Massachusetts, USA	ab3778	1:1000
COL2	ABclonal, Woburn, Massachusetts, USA	A1560	1:1000
β-Actin	Santa Cruz Biotechnology, Dallas, TX, USA	SC-47778	1:10000
p-ERK	Santa Cruz Biotechnology, Dallas, TX, USA	SC-7383	1:1000
ERK	Santa Cruz Biotechnology, Dallas, TX, USA	SC-1647	1:1000
p-JNK	Santa Cruz Biotechnology, Dallas, TX, USA	SC-6254	1:1000
JNK	Santa Cruz Biotechnology, Dallas, TX, USA	SC-7345	1:1000
p-p38	Santa Cruz Biotechnology, Dallas, TX, USA	SC-166182	1:1000
p38	Santa Cruz Biotechnology, Dallas, TX, USA	SC-271120	1:1000

Table 3: The pharmacological inhibitor applied in this study

Inhibitors	Target	Source	Catalog no	Working concentrations
FR180204	ERK	Santa Cruz Biotechnology, Dallas, TX, USA	SC-203945	10 µM
SP600125	JNK	Enzo Life Sciences, Inc. 10 Executive Blvd Farmingdale, NY 11735	BML-EI305-0010	10 µM
SB203580	p38	Enzo Life Sciences, Inc. 10 Executive Blvd Farmingdale, NY 11735	BML-EI286-0001	10 µM

Table 4: The pharmacological siRNA applied in this study

siRNA	Source	Catalog no	Working concentrations
ERK siRNA	Santa Cruz Biotechnology, Dallas, TX, USA	sc-35335	10 nM
JNK siRNA	Santa Cruz Biotechnology, Dallas, TX, USA	sc-29380	10 nM
p38 siRNA	Santa Cruz Biotechnology, Dallas, TX, USA	sc-29433	10 nM

Table 5: The correlation between has-miR-3074-5p to ERK, JNK, and p38 MAPK genes by using TargetScanHuman prediction tool (https://www.targetscan.org/vert_80/)

<table>
<tr><th>Binding position</th><th>Predicted consequential pairing of target region (top) and miRNA (bottom)</th><th>Site type</th><th>Context++ score percentile</th></tr>
<tr><td>Position 8185-8191 of MAPK1 3' UTR

hsa-miR-3074-5p</td><td>5' ...ACUGUCUGGUCGGCAGCAGGAAG...
|||||||
3' GACCGAGUCAAGUCGUCCUUG</td><td>7mer-m8</td><td>68</td></tr>
<tr><td>Position 3063-3070 of MAPK8 3' UTR

hsa-miR-3074-5p</td><td>5' ...AUAGUGAAAUCAUCA--GCAGGAAA...
||| |||||||
3' GACCGAGUCAAGUCGUCCUUG</td><td>8mer</td><td>81</td></tr>
<tr><td>Position 7566-7572 of MAPK14 3'UTR

hsa-miR-3074-5p</td><td>5' ...ACUGAGGAGGCUGAGGCAGGAAG...
|||||||
3' GACCGAGUCAAGUCGUCCUUG</td><td>7mer-m8</td><td>68</td></tr>
</table>
